# Supplementary material for: Differences in subjective well-being between individuals with distinct Joint Personality (temperament-character) networks in a Bulgarian sample
Source: PeerJ. 2022 Aug 26;10:e13956. doi: 10.7717/peerj.13956 (PMC9422977; doi:10.7717/peerj.13956)
Supplement: Supplemental Information 2 [file peerj-10-13956-s002.pdf]

**SUPPLEMENTARY MATERIAL**

# Differences in Subjective Well-Being between Individuals with Distinct Joint Personality (Temperament-Character) Networks in a Bulgarian Sample

Danilo Garcia[1, 2, 3, 4, 5*], Maryam Kazemitabar[6, 7], Kristina Stoyanova[8], Drozdstoj Stoyanov[8*], C. Robert Cloninger[5, 6, 9]

[1]Department of Behavioral Sciences and Learning, Linköping University, Linköping, Sweden

[2]Centre for Ethics, Law and Mental Health (CELAM), University of Gothenburg, Sweden

[3]Department of Psychology, Lund University, Lund, Sweden

[4]Promotion of Health and Innovation (PHI) Lab, International Network for Well-Being, Sweden

[5]Department of Psychology, University of Gothenburg, Gothenburg, Sweden

[6]Promotion of Health and Innovation (PHI) Lab, International Network for Well-Being, USA

[7]Yale School of Public Health, New Haven, Connecticut, USA

[8]Department of Psychiatry and Medical Psychology, Faculty of Medicine, Medical University, Plovdiv, Bulgaria

[9]Center for Well-being, Washington University School of Medicine in St. Louis, St. Louis, Missouri, USA

*Correspondence regarding this article should be addressed to D. Garcia, danilo.garcia@icloud.com, or D. Stoyanov, stojanovpisevski@gmail.com.

**Latent Profile Analyses (LPA): Temperament Profiles**

We examined four temperament models by investigating their fit indices. The optimum values for entropy and BIC belonged to Models 3 and the best values for AIC and SABIC to Model 4. However, the probability plots and outputs revealed that, except for Model 2, the models resulted in profiles with very few individuals (less than 5% of individuals). Model 2 included profile 1 (18.2% of the participants) with individuals who reported relatively low Novelty Seeking (n), high Harm Avoidance (H), low Reward Dependence (r), and low Persistence (p); and profile 2 (81.8% of the participants) with individuals who reported relatively low Novelty Seeking (n), low Harm Avoidance (h), relatively high Reward Dependence (R), and high Persistence (P). Table S1 presents the 4 models we tested. Figure S1 displays the plot of probabilities for Model 2, the chosen model, for Temperament Profiles.

Table S1. Fit indices for Temperament Profiles.

| Models | AIC | BIC | SABIC | Entropy | VLMRT | LMRT | BLRT |
|---|---|---|---|---|---|---|---|
| 1 | 5040.714 | 5073.462 | 5048.074 | | | | |
| 2 | 4985.190 | 5038.406 | 4997.150 | 0.678 | 0.0885 | 0.0939 | < .001 |
| 3 | 4960.442 | 5034.126* | 4977.002 | 0.684* | 0.0708 | 0.0755 | < .001 |
| 4 | 4945.496* | 5039.648 | 4966.656* | 0.635 | 0.8904 | 0.8930 | < .001 |

Note: * = optimum values for fit indices. The model number also refers to the number of Temperament Profiles in the model.

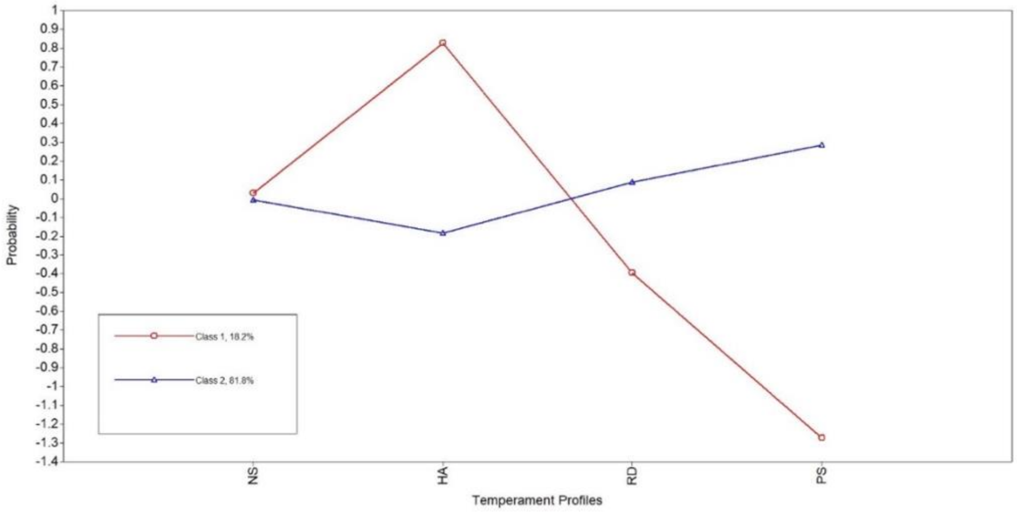


Figure S1. Plot of probability for the Temperament Profiles for Model 2.
Note: NS = Novelty Seeking, HA = Harm Avoidance, RD = Reward Dependence, PS = Persistence.

***Differences in Temperament Dimensions between and within Temperament Profiles***

Individuals in both Temperament Profiles reported low levels of Novelty Seeking (n = low Novelty Seeking) and were almost symmetrically different regarding high/low Harm Avoidance (H = high Harm Avoidance/h = low Harm Avoidance), high/low Reward Dependence (R = high Reward Dependence/r = low Reward Dependence), and high/low Persistence (P = high Persistence/p = low Persistence). To investigate this further, we used a one-way MANOVA to measure differences in temperament dimensions between individuals with distinct Temperament Profiles. We found significant differences between profiles in Harm Avoidance, Reward Dependence, and Persistence ($p$ < .001). The differences in Novelty Seeking between individuals were not significant ($p$ > .05), see Table S2 for the details.

Table S2. Measurement of differences in temperament dimensions between Temperament Profiles

| **One-way MANOVA** | | | | | |
|---|---|---|---|---|---|
| **Wilk's Lambda** | **Value** | **$F_{(4, 438)}$** | **Sig** | **$\eta^2$p** | **Observed power** |
| | .537 | 94.325 | <.001 | .463 | 1.000 |
| Test of between-subject effects | | | | | |
| **Temperament Dimensions** | **Mean square** | **F** | **Sig** | **$\eta^2$p** | **Observed power** |
| Novelty Seeking | .039 | .038 | .845 | .000 | .054 |
| Harm Avoidance | 79.316 | 96.435 | <.001 | .179 | 1.000 |
| Reward Dependence | 15.515 | 16.041 | <.001 | .035 | .979 |
| Persistence | 181.621 | 307.640 | <.001 | .411 | 1.000 |

In addition, two repeated measures MANOVA, one for each Temperament Profile, showed that there were significant differences in temperament dimensions within Temperament Profiles 1 and 2 ($p$ < .001). The post hoc tests with Bonferroni correction showed that for individuals with Temperament Profile 1, Harm Avoidance was significantly higher and that Persistence was significantly lower compared to their levels in any of the other temperament dimensions. However, within this Temperament Profile, scores in Novelty Seeking and Reward dependence were equally low ($p$ > .05). Conversely, individuals clustered in Temperament

Profile 2 had significantly lower scores in Harm Avoidance than in Persistence and higher scores in Reward Dependence than in Harm Avoidance. Mean differences between individuals' scores in Novelty Seeking and in Harm Avoidance as well as in Novelty Seeking and in Reward Dependence were not significant ($p > .05$) within Temperament Profile 2 (see Figure S2).

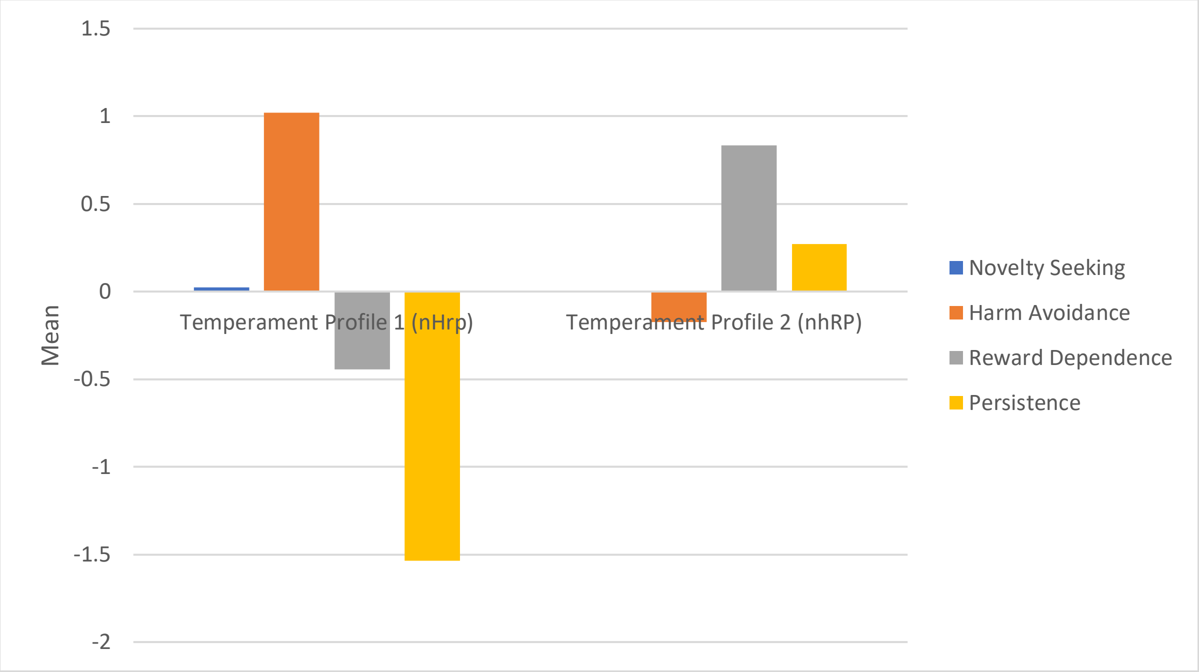


Figure S2. Differences in temperament dimensions (*z-scores*) between and within Temperament Profiles.

Note. n = low Novelty Seeking, H = high Harm Avoidance, h = low Harm Avoidance, R = high Reward Dependence, r = low Reward Dependence, P = high Persistence, p = low Persistence.

### *The Temperament Profiles*

Individuals in both Temperament Profiles reported low levels of Novelty Seeking (n) but were symmetrically different regarding high/low Harm Avoidance (h/H), high/low Reward Dependence and high/low Persistence (P/p). Hence suggesting that individuals in Temperament Profile 1 (nHrp) might be described as inhibited (nH), aloof (Hr), privacy-seeking (nr), and having difficulties to initiate anything new because of their inhibitions rooted in their tendency to pragmatism and underachievement (p). They are methodical in the sense that they are highly cautious (H), orderly (n), and objective (Hr). If such individual lacks a well-developed

Character Profile, they can be perceived and act as obsessional personalities and find situations that require exposure to public attention to be challenging (Cloninger. 2004). They are, however, not afraid of being rejected (Hr), hence, making them objective. Hence, we labeled this profile as Methodical (nHrp). Individuals in Temperament Profile 2 might be described as Reliable (nhRP) because they are stable (nh), warmly sociable (hR), traditional (nR), and hard-working (P). Hence, it is highly likely that they can be trusted to carry out what they are expected to do in a predictable and traditional manner and to develop a mature character (Cloninger, 2004).

***Differences in Subjective Well-Being between Individuals with Distinct Temperament Profiles***

We investigated mean differences in subjective well-being between individuals with distinct Temperament Profiles using a MANOVA, which revealed significant differences (*Wilks' Lambda* = .86, $F_{(3, 439)} = 24.328$, $p < .001$, $\eta^2 p = .143$). The test of between-subject effects showed that positive affect, negative affect, and life satisfaction were significantly different between Temperament Profiles ($p < .001$). Moreover, a post hoc test with Bonferroni correction showed that positive affect and life satisfaction were higher among individuals with a Reliable (nhRP) Temperament Profile and negative affect was higher among individuals with a Methodical (nHrp) Temperament Profile ($p < .005$).

**Latent Profile Analyses (LPA): Character Profiles**

As for the Temperament Profiles, for the Character Profiles, we tested four models using LPA. We found that the optimum values for BIC and entropy pertained to Models 3 and AIC and SABIC to Model 4. VLMRT, LMRT, and BLRT best significant values belonged to Model 2. The investigation of the probability plots demonstrated that Model 2 was the best model that fitted to our data (see Figure S3), since Models 3 and 4 had profiles with insufficient number of individuals (i.e., less than 5%). This model consisted of profile 1 which consisted of 23.1%

of participants who reported low Self-Directedness (s), low Cooperativeness (c), and low Self-Transcendence (t); and profile 2 with 76.9% of the participants who reported high Self-Directedness (S), high Cooperativeness (C), and relatively low Self-Transcendence (t). Table S3 shows the 4 models tested for profiling individuals using their character scores. Figure S3 displays the probability plot for Model 2, the chosen model, for the Character Profiles.

Table S3. Fit indices for character dimension profiles

| Model | AIC | BIC | SABIC | Entropy | VLMRT | LMRT | BLRT |
|---|---|---|---|---|---|---|---|
| 1 | 3780.535 | 3805.097 | 3786.055 | | | | |
| 2 | 3643.293 | 3684.229 | 3652.494 | 0.776 | < .001 | < .001 | < .001 |
| 3 | 3605.458 | 3662.768* | 3618.338 | 0.812* | 0.0102 | 0.0121 | < .001 |
| 4 | 3600.285* | 3673.969 | 3616.845* | 0.745 | 0.3660 | 0.3769 | 0.0714 |

Note: * = optimum values for fit indices. The model number also refers to the number of Character Profiles in the model.

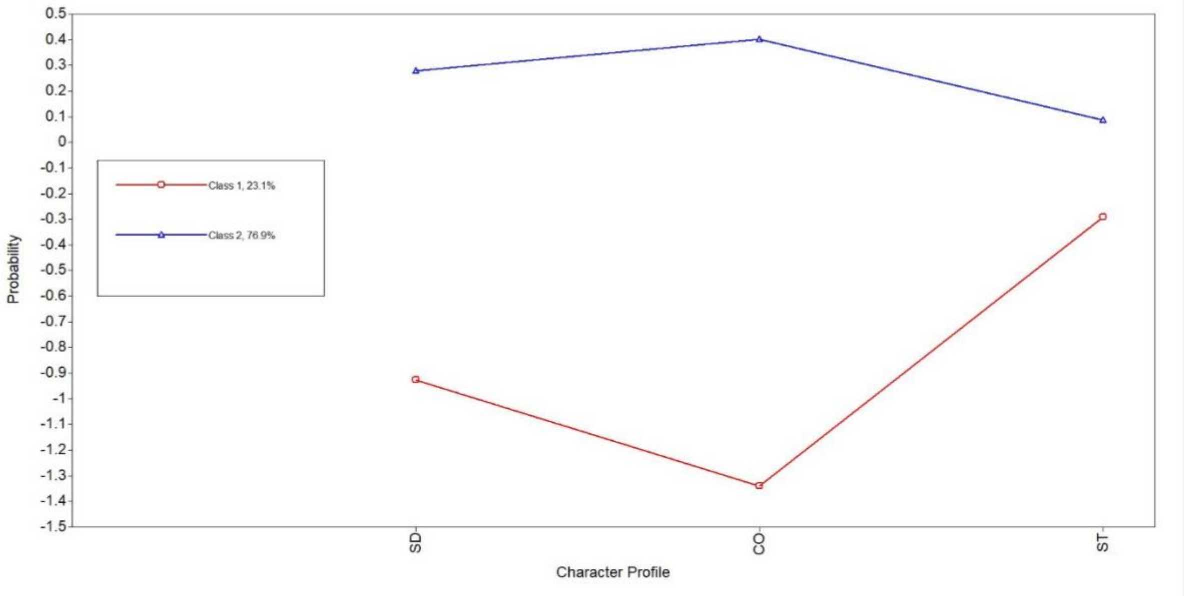


Figure S3. Plot of probability for the Character Profiles for Model 2.
Note. SD = Self-Directedness, CO = Cooperativeness, ST = Self-Transcendence.

***Differences in Character Dimensions between and within Character Profiles***

Individuals in the Character Profiles 1 and 2 were symmetrically different regarding high/low Self-Directedness (S = high Self-Directedness/s = low Self-Directedness), high/low Cooperativeness (C = high Cooperativeness /c = low Cooperativeness) and high/low Self-Transcendence (T = high Self-Transcendence/t = low Self-Transcendence). To investigate this

further, we used a one-way MANOVA and found that Self-Directedness, Cooperativeness, and Self-Transcendence were significantly higher among individuals with Character Profile 2 compared to Character Profile 1 ($p < .001$). Nevertheless, individuals in both profiles had low levels of Self-Transcendence. See Table S4 and Figure S4 for the details.

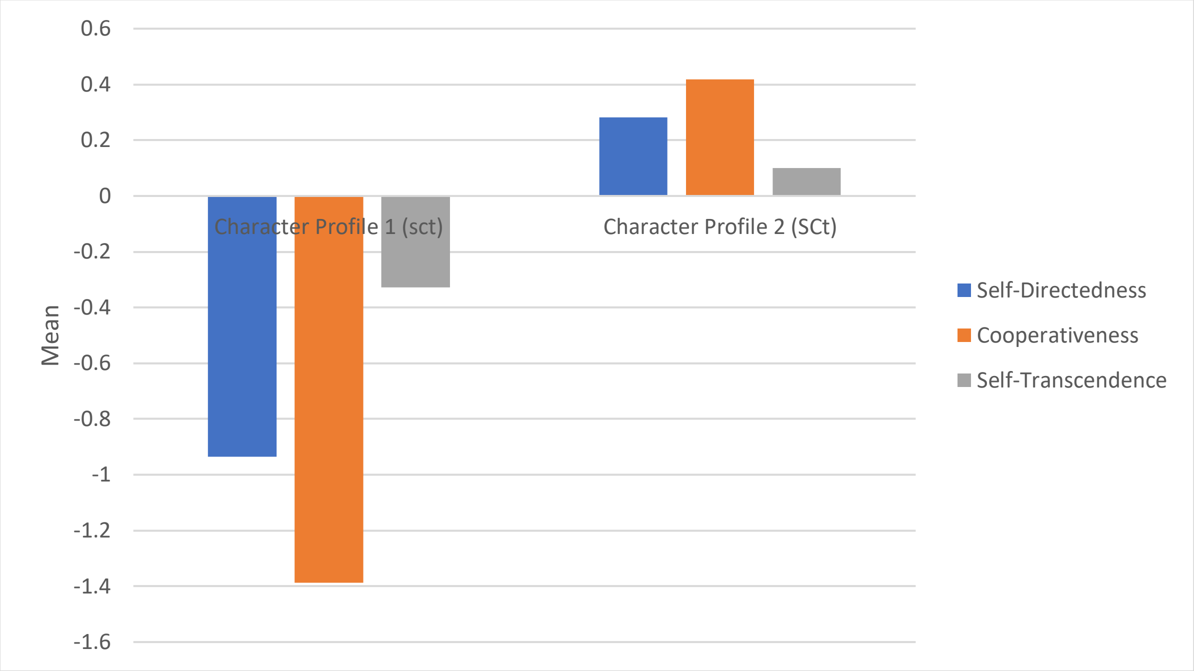


Figure S4. Differences in character dimensions (*z-scores*) between and within Character Profiles.
Note. S = high Self-Directedness, s = low Self-Directedness, C = high Cooperativeness, c = low Cooperativeness, t = low Self-Transcendence.

Table S4. Measurement of differences in character dimensions between Character Profiles.

| **One-way MANOVA** | | | | | |
|---|---|---|---|---|---|
| **Wilk's Lambda** | **Value** | **$F_{(3, 439)}$** | **Sig** | **$\eta^2p$** | **Observed power** |
| | .393 | 226.295 | .000 | .607 | 1.000 |
| Test of between-subject effects | | | | | |
| **Character Dimensions** | | **Mean square** | **F** | **Sig** | **$\eta^2p$** |
| Self-directedness | | 114.787 | 154.716 | < .001 | .260 |
| Cooperativeness | | 251.958 | 584.730 | < .001 | .570 |
| Self-transcendence | | 14.137 | 14.571 | < .001 | .032 |

In addition, a repeated measures MANOVA showed that for Character Profile 1, all character dimensions were significantly distinct from each other ($p < .001$) and that Self-

Transcendence had the highest and Cooperativeness the lowest levels in this Character Profile. A second repeated measures MANOVA showed that for Character Profile 2, Self-Directedness and Self-Transcendence levels were not significantly different ($p > .05$) and that Cooperativeness had the highest level in this Character Profile.

***The Character Profiles***

The analyses suggested that all individuals in this Bulgarian population were low in Self-Transcendence (t). Individual who are low in Self-Transcendence are described as individualistic, skeptical, conventional, and cynical (Cloninger, 2004). The individuals in Character Profile 1 might be described as Apathetic (sct). Individuals with this type of character report the lowest levels of overall well-being and health (Cloninger, 2004). They report experiencing unhealthy emotions such as anxiety, alienation and have high rates of mental and physical disorders. Indeed, individuals with an Apathetic Character Profile feel victimized and helpless (sc), show very poor judgement (st) and are distrustful (ct). In other words, they experience the world from an outlook of separateness, which leads to fear, excessive desire, and false pride or self-reproach. The individuals in Character Profile 2 might be described as Organized (SCt) (Cloninger, 2004). They are often perceived as mature leaders (SC), logical (St), and conventional (Ct). They are, most of the time, happy and healthy, and seldom need health care. However, when they face difficult existential challenges, such as, severe illness or death, they often lack the necessary outlook of unity and connectedness needed to be resilient through such situations (t).

***Differences in Subjective Well-Being between Individuals with Distinct Character Profiles***

We investigated mean differences in subjective well-being between individuals with distinct Character Profiles using a MANOVA, which revealed significant differences (*Wilks' Lambda* = .97, $F_{(3, 439)} = 4.36$, $p \leq .005$, $\eta^2 p = .03$). The test of between-subject effects showed that negative affect and life satisfaction differed significantly between individuals with distinct

Character Profiles ($p < .05$) while positive affect were equal between them ($p > .05$). A post hoc test with Bonferroni correction showed that life satisfaction was higher among individuals with the Organized (SCt) Profile and negative affect was higher among individuals with the Apathetic (sct) Profile ($p < .001$). Positive affect, however, did not differ between individuals with these two Character Profiles.

**Relationships between Temperament Profiles and Character Profiles in the Joint Personality (temperament-character) Networks**

We examined the relationships between Temperament Profiles and Character Profiles within each one of the two Joint Personality Networks. Table S5 and S6 depicts the crosstabulation between Temperament Profiles and Character Profiles in Joint Personality Network 1 and 2, respectively. In the Joint Personality Network 1, 100% of individuals had a Reliable (nhRP) Temperament Profile in combination with an Organized (SCt) Character Profile. In the Joint Personality Network 2, 18.7% of individuals had a Methodical (nHrp) Temperament Profile in combination with an Apathetic (sct) Character Profile, 28.1% had a Methodical (nHrp) Temperament Profile in combination with an Organized (SCt) Character Profile, 53.2% had a Reliable (nhRP) Temperament Profile in combination with an Apathetic (sct) Character Profile. In sum, all individuals in the Joint Personality Network 1 had a stable Reliable Temperament Profile in combination with an Organized Character Profile, while in the Joint Personality Network 2 none of the individuals had this stable and organized Temperament and Character Profile combination. Instead 71.9% had an Apathetic (sct) Character Profile in combination with a Methodical (nHrp) or a Reliable (nhRP) Temperament Profile and 28.1% had an Organized (SCt) Character Profile in combination with a Methodical (nHrp) Temperament Profile. Figure S5 shows the membership of each Temperament Profile and Character Profile within each Joint Personality Network.

Table S5. Crosstabulation of the Temperament Profiles and Character Profiles in Joint Personality Network 1.

| | | | **Character Profiles** | |
|---|---|---|---|---|
| | | | **Organized (SCt)** | **Total** |
| **Temperament Profiles** | **Reliable (nhRP)** | Count | 304 | 304 |
| | | % within Temperament Profile | 100.0% | 100.0% |
| | | % of Total | 100.0% | 100.0% |

Note: n = low Novelty Seeking, h = low Harm Avoidance, R = high Reward Dependence, P = high Persistence, S = high Self-Directedness, C = high Cooperativeness, t = low Self-Transcendence.

Table S6. Crosstabulation of the Temperament Profiles and Character Profiles in Joint Personality Network 2.

| | | | **Character Profiles** | | |
|---|---|---|---|---|---|
| | | | **Apathetic (sct)** | **Organized (SCt)** | **Total** |
| **Temperament Profiles** | **Methodical (nHrp)** | Count | 26 | 39 | 65 |
| | | % within Temperament Profile | 40.0% | 60.0% | 100.0% |
| | | % of Total | 18.7% | 28.1% | 46.1% |
| | **Reliable (nhRP)** | Count | 74 | 0 | 74 |
| | | % within Temperament Profile | 100.0% | 0.0% | 100.0% |
| | | % of Total | 53.2% | 0.0% | 53.2% |
| **Total** | | Count | 100 | 39 | 139 |
| | | % within Temperament Profile | 71.9% | 28.1% | 100.0% |
| | | % of Total | 27.7% | 72.3% | 100.0% |

Note: n = low Novelty Seeking, H = high Harm Avoidance, h = low Harm Avoidance, R = high Reward Dependence, r = low Reward Dependence, P = high Persistence, p = low Persistence, S = high Self-Directedness, s = low Self-Directedness, C = high Cooperativeness, c = low Cooperativeness, t = low Self-Transcendence.

**References**


Cloninger, C.R. (2004). *Feeling good: The science of wellbeing*. New York: Oxford University Press.

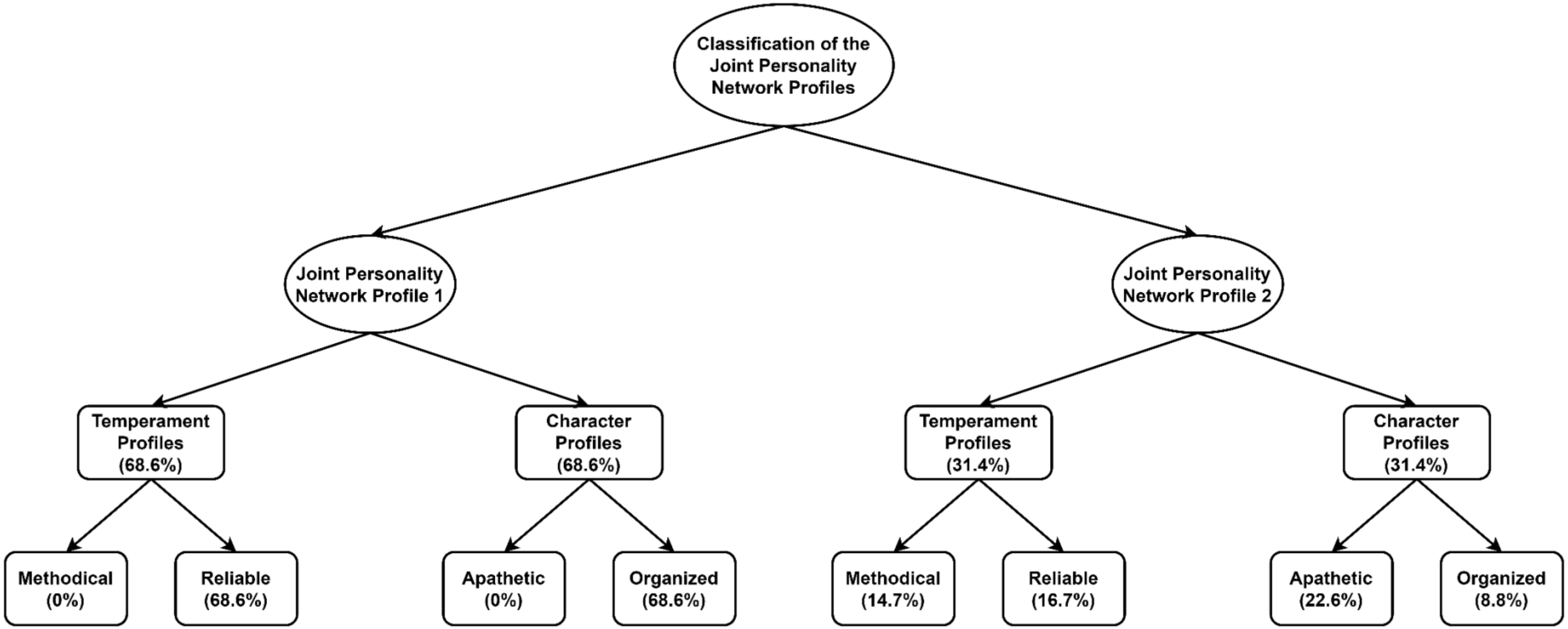


Figure S5. Joint Personality Network membership.
